# New words in human mutagenesis

**DOI:** 10.1186/1471-2105-12-268

**Published:** 2011-06-30

**Authors:** Alexander Y Panchin, Sergey I Mitrofanov, Andrei V Alexeevski, Sergey A Spirin, Yuri V Panchin

**Affiliations:** 1 Department of Bioengineering and Bioinformatics, Moscow State University, Vorbyevy Gory 1-73, Moscow, 119992, Russian Federation; 2 Institute for Information Transmission Problems, Russian Academy of Sciences, Bolshoi Karetny pereulok 19-1, Moscow, 127994, Russian Federation; 3 Department of Mathematical Methods in Biology, Belozersky Institute, Moscow State University, Vorbyevy Gory 1-40, Moscow, 119991, Russian Federation; 4 Department of Mathematics, Scientific-Research Institute for System Studies, Russian Academy of Sciences, Nakhimovskii prospekt 36-1, Moscow, 117218, Russian Federation

## Abstract

**Background:**

The substitution rates within different nucleotide contexts are subject to varying levels of bias. The most well known example of such bias is the excess of C to T (C > T) mutations in CpG (CG) dinucleotides. The molecular mechanisms underlying this bias are important factors in human genome evolution and cancer development. The discovery of other nucleotide contexts that have profound effects on substitution rates can improve our understanding of how mutations are acquired, and why mutation hotspots exist.

**Results:**

We compared rates of inherited mutations in 1-4 bp nucleotide contexts using reconstructed ancestral states of human single nucleotide polymorphisms (SNPs) from intergenic regions. Chimp and orangutan genomic sequences were used as outgroups. We uncovered 3.5 and 3.3-fold excesses of T > C mutations in the second position of ATTG and ATAG words, respectively, and a 3.4-fold excess of A > C mutations in the first position of the ACAA word.

**Conclusions:**

Although all the observed biases are less pronounced than the 5.1-fold excess of C > T mutations in CG dinucleotides, the three 4 bp mutation contexts mentioned above (and their complementary contexts) are well distinguished from all other mutation contexts. This provides a challenge to discover the underlying mechanisms responsible for the observed excesses of mutations.

## Background

A cytosine followed by a guanine (CG) is the best known example of a nucleotide word within the human genome that has a dramatically increased probability to undergo mutation [1]. By the early 1960s, researchers already knew that many animal genomes have a deficit in CG dinucleotides [2,3]. This was later explained by DNA methylation. Specific DNA methyltransferase enzymes convert cytosines in CG dinucleotides into methyl-cytosines, which are susceptible to deamination to thymine [4]. This mutation mechanism is involved in cancer development [5], has shaped the composition of our genome [6,7], and remains profoundly interesting to molecular and evolutionary biologists today [8].

Other sequences within the genome are subject to biases of varying magnitude [9-12], and knowledge of these biases has contributed greatly to our understanding of the molecular mechanisms involved in mutagenesis [13,14]. A nucleotide context affects not only the rate of substitution, but also the rates of deletions and insertions [15]. Mutation rates within sequences are also influenced by local CpG content [16], are dependent on the chromosome on which the sequences is located [17,18], and vary between different regions on the same chromosome [19,20]. Additionally, excessive amounts of mutations have been reported in certain repeated elements [21]. The combination of different mutation factors and selection has led to the existence of mutation hot-spots [10].

With the exception of the CG sequence, relationships between substitution rates and neighbouring nucleotides in the human germline are still poorly understood. To the best of our knowledge, no other sequences have been shown to influence the rates of inheritable mutations in a way comparable to the effect of CG dinucleotides on C > T transition rates. The goal of the present study was to identify such sequences. For such a study, the ideal would be to analyze the genome sequences of parents and their children as a direct source of mutation data. However, such data are currently unavailable. Therefore, we used a dataset of presumably neutral mutations that occurred in the human lineage after the separation of humans and chimpanzees. Such data can be derived from human intergenic SNPs for which the ancestral state has been reconstructed.

We show that the mutation rates within three 4 bp nucleotide contexts in the human genome (and their complementary contexts) stand out from the mutation rates of all other 2-4 bp mutation contexts. The effects of these contexts on mutation rates is comparable to the effect of CG nucleotides on C > T mutation rates.

## Results

To study mutation processes separately (as far as possible) from the effects of natural selection, we used human single nucleotide polymorphisms (SNPs) from regions that do not belong to any known genes or CpG islands, and are not within 1000 bp of flanking regions of known genes. A genomic polymorphism in the human population can be attributed, in the vast majority of cases, to a relatively recent mutation. Theoretically, a polymorphism may be inherited from a common ancestor of human and apes, but the proportion of such polymorphisms seems to be relatively small [10]. Indeed, most of them should be rare cases of stabilizing selection in favour of the polymorphism. Thus, it is possible to compare each human SNP with the corresponding nucleotides in the genomic sequences of apes, namely chimpanzee (*Pan troglodytes*) and orangutan (*Pongo pygmaeus*). In cases where both ape nucleotides coincide with one of the human SNP variants, we can identify the ancestral state of the SNP, i.e., the direction of the mutation. Using this method, we identified the direction of 3,405,095 probable mutations. This mutation data are available to download at: http://mouse.belozersky.msu.ru/SNP/. UCSC Human Genome Browser mapping of human SNPs to chimp and orangutan genomes was used [22]. This amount of data was judged to be sufficient for the analysis of 1-4 bp mutation contexts.

We used a measure called 'contrast' to evaluate if the addition of specific nucleotides to the 5' or 3' end of 1-3 bp words increases the probability of observing certain mutations in fixed positions (see Methods). For example, there is a 5.1-fold excess of C to T (C > T) mutations if C is followed by G, when compared with the rate of C > T mutations on average in the genome. We say that the mutation context description is {C > T|1, CG}, and that it has a contrast of 5.1 when compared with its {C > T|1, C} subcontext. Contrast values higher than 1 represent an excess of mutations, while values smaller than 1 represent mutation deficiency. Contrast values for a mutation context {mut|pos, W} and subcontext {mut|pos', W'} are computed based on the occurrences of words W and W' and the number of mutations observed in {mut|pos, W} and {mut|pos', W'}, respectively (see Methods). To avoid misunderstanding, note that our concept of contrast is not related to the contrast between the number of samples used in variance analysis.

Each mutation context can be characterized by two contrast values: mutation bias and minimal contrast. The minimal contrast value of a context is its contrast value closest to 1 among all contrast values, when compared with each of the contexts subcontexts. For example, the {C > T|2, ACG} context has three such subcontexts {C > T|2, AC}, {C > T|1, CG}, and {C > T|1, C} giving contrast values of 5.08, 1.08 and 5.48, respectively. 1.08 is the minimal contrast for {C > T|2, ACG}. Contrast values obtained using one-letter subcontexts such as {C > T|1, C} are called mutation biases. The value 5.48 is the mutation bias for {C > T|2, ACG} because there is a 5.48-fold excess of C > T mutations in position two of the word ACG, when compared with the average C > T mutation rate in the genome.

Mutation bias indicates the total excess (or deficiency) of mutations within a given context. Minimal contrast indicates the excess (or deficiency) of mutations within a given context that cannot be explained by the excess (or deficiency) of mutations in one of its subcontexts, thus representing the actual role of the context as a whole. For dinucleotide contexts such as {C > T|1, CG}, mutation bias equals minimal contrast.

**Figure 1:**
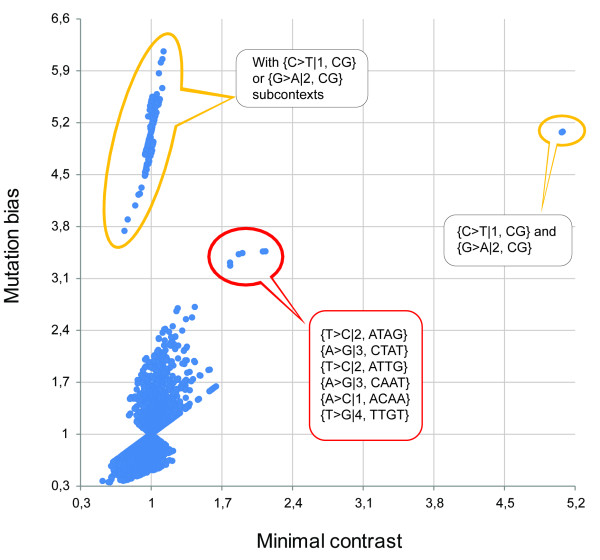
**Two-dimensional plot of mutation bias versus minimal contrast**. Two-dimensional plot of mutation bias versus minimal contrast of all 1-4 bp mutation contexts. Several exclusive clusters are outlined. The small orange cluster contains {C > T|1,CG} and {G > A|2,CG} contexts. It can be discerned by minimal contrast from the large orange cluster that contains all 3-4 bp contexts with {C > T|1,CG} and {G > A|2,CG} subcontexts and only such contexts. The red cluster contains six 4 bp contexts distinguishable by both minimal contrast and mutation bias.

Figure 1 contains a two-dimensional plot of mutation bias *versus *minimal contrast; each dot represents a mutation context. The current analysis does not allow us to discern the strand of DNA on which a mutation occurred. This is why each mutation context has a complementary context with similar mutation properties: dots are situated on the plot in pairs. On the plot, besides the large cluster that includes the majority of contexts, we can see three distinct small clusters. As would be expected, the cluster characterized by the highest value of minimal contrast consists of two dots, representing the {C > T|1, CG} context and its complementary {G > A|2, CG} context. A second cluster is characterized by low minimal contrast and high mutation bias, and consists of all contexts, and only such contexts, that have {C > T|1, CG} or {G > A|2, CG} subcontexts. Finally, there is a distinct cluster that is characterized by both high mutation bias and minimal contrast. It contains three pairs of mutation contexts. These contexts are {T > C|2, ATTG} and its complement {A > G|3, CAAT}; {T > C|2, ATAG} and {A > G|3, CTAT}; and {A > C|1, ACAA} and {T > G|4, TTGT}.

**Figure 2:**
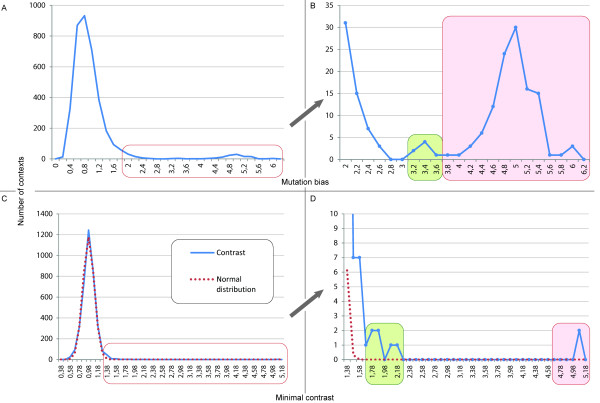
**Histogram of mutation biases and minimal contrasts**. Histogram of mutation biases (Figures 2A and 2B) and minimal contrasts (Figure 2C and 2D). Figures 2A and 2C are complete histograms. Figures 2B and 2D are enlarged fragments of the outlined areas of figures 2A and 2C, respectively. 2B: red selection contains only those contexts that have {C > T|1, CG} and {G > A|2, CG} subcontexts and the contexts {C > T|1, CG} and {G > A|2, CG} themselves. 2D: the red selection contains only the {C > T|1, CG} and {G > A|2, CG} contexts. The brown selections in figures 2B and 2D contain only the following contexts: {T > C|2, ATTG} and complementary {A > G|3, CAAT}, {T > C|2, ATAG} and {A > G|3, CTAT}, {A > C|1, ACAA} and {T > G|4, TTGT}. 2C, 2D: red dots show a normal distribution (expectation 0.99, standard deviation 0.12).

On Figure 2, the distributions of mutation bias and minimal contrast values for all 1-4 bp contexts are shown. The enlarged fragment (Figure 2B) demonstrates that the contexts {T > C|2, ATTG} and {A > G|3, CAAT}, {T > C|2, ATAG} and {A > G|3, CTAT}, {A > C|1, ACAA} and {T > G|4, TTGT}, along with {C > T|1, CG}- and {G > A|2, CG}-containing contexts are well distinguished by their mutation biases. Additionally, it can be noted that all 5 bp mutation contexts containing {T > C|2, ATTG}, {A > G|3, CAAT}, {T > C|2, ATAG}, {A > G|3, CTAT}, {A > C|1, ACAA}, and {T > G|4, TTGT} subcontexts also have high mutation bias values (data not shown). The eight contexts{C > T|1, CG} and {G > A|2, CG}; {T > C|2, ATTG} and {A > G|3, CAAT}; {T > C|2, ATAG} and {A > G|3, CTAT}; {A > C|1, ACAA} and {T > G|4, TTGT} also have the highest minimal contrast values (Figure 2D).

It is difficult to predict the distribution of mutation biases and minimal contrasts theoretically, because of the dependence of contexts: one mutation from the data set is considered in all possible contexts of 1-4 bp length, which are clearly not independent. Indeed, it is unclear how to approximate the distribution of mutation biases (Figure 2A). Surprisingly, in the range 0.7 - 1.2 the distribution of minimal contrast values can be approximated by the normal distribution with the mean of 0.99 and a sigma value of 0.12 (Figure 2C). In the range 1.2 - 5.1 these distributions are significantly different at *p *< 0.001 (Figure 2D). If we assume that mutations are random and that the normal distribution reflects the distribution of minimal contrast values, then over one hundred 1-4 bp contexts may significantly influence mutation rates. The eight mentioned contexts are the most extreme among them.

There are 3.5 and 3.3-fold excesses of T > C mutations in position two of ATTG and ATAG words, respectively, suggesting that a highly mutable pattern AT[A/T]G (ATWG) exists in the human genome (note that complementary contexts behave in the same way). There is also a 3.4-fold excess of A > C mutations in position one of the word ACAA. These effects are comparable to the 5.1-fold excess of C > T mutations in the first position of the CG dinucleotide. The existence of excessive mutations in each of the described contexts compared with any of their subcontexts is statistically significant at *p *< 10^-15^, taking the Bonferroni correction into account. Our main results did not change when we used SNPs from the whole human genome (6,530,908 SNPs) (Table 1). We also found 2890 (out of 3744) other contexts with mutation rates significantly different at *p *< 10^-15 ^from the mutation rates of each of their subcontexts. However, here we would like to concentrate on the most extreme cases, which are comparable with {C > T|1, CG} in terms of contrast values and mutation biases.

**Table 1:** Mutation contexts

Mutation type	Subcontext	Context	Minimal contrast		Mutation bias	
			**(NG)**	**(WG)**	**(NG)**	**(WG)**

C > T	**C**	**C**G	5.1	4.7	5.1	4.7
G > A	**G**	C**G**	5.1	4.7	5.1	4.7
T > C	**T**TG	A**T**TG	2.1	2.2	3.5	3.6
T > C	A**T**A	A**T**AG	1.8	1.8	3.3	3.5
A > G	CA**A**	CA**A**T	2.1	2.2	3.5	3.6
A > G	T**A**T	CT**A**T	1.8	1.8	3.3	3.5
A > C	**A**CA	**A**CAA	1.9	1.9	3.4	3.4
T > G	TG**T**	TTG**T**	1.9	1.9	3.5	3.4

This table presents contexts of mutations that have minimal contrast values higher than 1.7 and mutation bias values higher than 3. The mutated nucleotides are in bold and underlined. "NG" stands for "No Genes" (regions that do not belong to any known genes, CpG islands, or 1000 bp flanking regions of known genes) and "WG" stands for "Whole Genome". The subcontext is the one that is used for the minimal contrast calculation and is the same for the no gene and whole genome datasets for all mentioned contexts.

## Discussion

In our study, we used parsimony to infer the ancestral states of the SNPs. However, there is a risk of misinterpretation when using parsimony and dealing with rapid mutations. For example, it has been stressed that if the human sequence is CG, the chimp sequence is TG, and a TG sequence from the baboon is used as an outgroup, the human and chimp ancestral sequence is actually more likely to be CG, not TG as parsimony would predict [23].This occurs because of highly elevated CG > TG mutation rates. However, in our study, instead of just one outgroup species, two outgroup species were used, and the ancestral state of two human SNP variants was inferred by comparing those variants with chimp and orangutan sequences. Any scenario alternative to the one estimated by parsimony would require at least three alternative mutations, not just two.

To reduce the effects of natural selection we analyzed only those DNA regions that are far away from the known genes. However, several studies have shown that selective constraints can be found in non-coding and even in intergenic regions of the human genome [24,25]. Also, regions subject to accelerated human evolution, possibly caused by positive selection, were found to be enriched in gene deserts (regions *>*500 kb without an Ensembl gene) [26]. Intergenic regions that were found to be subject to selective constraints or accelerated evolution were not specifically excluded from our analysis. We postulated that the total length of such regions would be too small to have a pronounced effect on the observed genome-wide mutation rates.

Substitution rate biases were recently studied by Nevarez et al. using a measure called relative abundance [27]. Relative abundance indicates whether mutations in a context happen at rates different from those expected from the mutation frequencies of all of that context's subcontexts, including discontigous ones (for example, ATNG). Although none of the mutation contexts that we have identified using the minimal contrast method were highlighted in that study as being exceptional, it is worth mentioning that the {T > C|2, ATTG} context has the highest relative abundance among all 4-bp mutation contexts. The {T > C|2, ATNG} discontigous context (a less specific representation of the {T > C|2, ATWG} motif we identified) also had high relative abundance. However, the {A > C|1, ACAA} context did not. Also, it is important to note that in the study by Nevarez et al, substitutions were studied, not SNPs. It can be argued that fixed substitutions should be, on average, under higher selective pressure than SNPs. SNPs are not fixed in the population, and are likely to be more neutral.

Before comparing relative abundance and minimal contrast, the two values used for measuring the influence of contexts on mutation rates, it should be noted that, at the moment, there is no universal statistical model that can adequately describe the frequencies of short nucleotide words in a genome. The lack of such a model can be demonstrated using the example of 3 bp word frequencies in the human genome. Previously, we found that all observed frequencies of 3 bp words significantly differ (*p *< 10^-6^) from the expected frequencies based on the frequencies of single nucleotides and 2 bp words, even with the best models among the many different models found in the literature [7].

The lack of a universally accepted statistical model of the genome has led to authors using different approximations that are most suitable for their research task. For a genome-scale comparison of word frequencies, we believe the Relative Abundance Value, suggested by Karlin et al. [28] to be the most balanced approximation. This value was used in a number of subsequent studies, including one of our own [7]. Relative abundance was modified to estimate the frequencies of mutations in nucleotide contexts in the article by Nevarez et al. [27]. These values should give a good approximation of the expected mutation frequencies, and their comparison with the observed frequencies should reveal the complete picture of a context's impact on mutation frequencies.

In the present study, we used different values - minimal contrast and mutation bias - because our goal was to find those contexts, besides {C > T|1,CG}), that have a dramatic effect on mutation rates. If context {mut|pos, w} is highly mutable because of the existence of some context-dependent mutation mechanism, then the number of mutations observed in this context will be significantly higher than expected from the mutation rates in no particular context (mutation bias) or in any of its subcontexts (minimal contrast). The distribution of mutation rates among subcontexts is not important in this case.

To produce a complete picture of the context's dependence of mutation frequencies, minimal contrast is, of course, not as good as relative abundance. For example, in cases when a context's contrast with one subcontext is -0.91 and for the other it is +1.09, the minimal contrast will be smaller or larger than 1 depending on the number in the third decimal position of the contrast values. This value is obviously unstable. However, this is not the case for any of the contexts that are highlighted in our article. Despite this problem, minimal context allowed us to reach our goal and identify several contexts that dramatically affect mutation rates.

As previously mentioned, the elevated frequency of mutations in the {C > T|1,CG} context in the human genome is consistent with the underrepresentation of CpG dinucleotides. It would not be surprising if the words ATTG, ATAG, and ACAA, which are present in the highly mutable contexts described in our study, would also be underrepresented in the human genome, and in the genomes of closely related species. Data on the underrepresentation and overrepresentation of 1-7 bp words in 139 complete genomes were recently published [7]. While ATTG seems to be slightly underrepresented in all 22 studied mammalian genomes (including the human genome), ATAG is underrepresented in only 13 mammalian genomes, and ACAA is actually slightly overrepresented in all but two mammalian genomes. This can be explained either by the novelty of the underlying mechanisms that lead to excessive mutations in the {T > C|2,ATTG}, {T > C|2,ATAG}, and {A > C|1,ACAA} contexts or by the fact that other mutations leading to the loss or accumulation of ATAG, ATTG, and ACAA words are not accounted for. It is also worth mentioning that none of the three mentioned mutation contexts have elevated minimal contrast or mutation bias values in *Drosophila melanogaster *(unpublished data).

Excessive mutations in the {T > C|2,ATTG}, {T > C|2,ATAG}, and {A > C|1,ACAA} contexts suggest the possible existence of underlying mechanisms in a similar way in which DNA methyltransferase activity is at least partially responsible for excessive mutations in the {C > T|1,CG} context.

## Conclusions

Three 4 bp mutation contexts with contrastingly high mutation rates exist in the human genome, suggesting the existence of previously unknown context-dependent molecular mechanisms involved in human mutagenesis and providing challenges for further experimental research. Two of these contexts can be combined into one highly mutable motif (AT[A/T]G). The excess of mutations in these contexts is not explained by excessive mutations in their subcontexts.

## Methods

### SNP inclusion criteria

Human SNPs (dbSNP 130) from regions aligned to chimpanzee and orangutan genomes, according to the UCSC Human Genome Browser [12], where obtained. To reduce bias from natural selection we excluded SNPs from any UCSC genes, CpG islands, or flanking regions within 1000 bp of UCSC genes. Coordinates of CpG islands were also taken from the UCSC Human Genome Browser. SNPs were included only if the following prerequisites were met:

(1) Exactly two human SNP variants are reported in the SNP database.

(2) One of the two human SNP variants is the same as the orthologous nucleotides in both chimp and orangutan genomes.

(3) 10 bp upstream and downstream regions adjacent to the human SNP (SNP regions) are aligned to the chimpanzee and orangutan genomes, as reported by the UCSC database, and the corresponding sequences (orthologous regions) in these genomes can be identified.

(4) The SNP regions and the corresponding orthologous regions in chimpanzee and orangutan genomes do not contain gaps or unknown nucleotides.

(5) The SNP regions differ by no more than by one substitution compared with the orthologous regions in chimpanzee (the SNP position is not taken into account).

(6) The SNP regions differ by no more than six substitutions compared with the orthologous regions in orangutan.

(7) Three nucleotides upstream, as well as three nucleotides downstream, of the SNP are the same in human and chimp genomes.

### Mutation context and subcontext

We denote the mutation context of mutation *mut *in position *pos *of the word *W *as {*mut*|*pos*, *W*}. For example, {C > T|1, CG} represents a C > T mutation in the first position of the word CG.

Mutation context {*mut*|*pos'*, *W'*} is called a subcontext of the context {*mut*|*pos*, *W*} if *W' *is a subword of *W *and any mutation *mut *occurring in position *pos *of the word *W *is at the same time a mutation occurring in position *pos' *of the word *W'*. For example, {C > T|1, CG} is a subcontext of {C > T|2, ACG}.

### Contrast

For each pair of context {*mut*|*pos*, *W*} and its subcontext {*mut*|*pos'*, *W'*} the value of contrast is given by the formula:

Here P{*mut*|*pos*, *W*} and P{*mut*|*pos'*, *W'*} are the conditional probabilities of observing mutation *mut *in the position *pos *of the word *W*, and position *pos' *of word *W'*, respectively, in a given dataset. Although these probabilities cannot be explicitly calculated without assumptions of the general probability of mutation per nucleotide in the genome, their ratio can be estimated by the following formula:

Here, P_*W *_and P_*W' *_are the observed frequencies of words W and W' respectively among all words of the same length in the following intervals adjacent to the human SNP: from position -10 to position -6 and from position 6 to position 10 inclusively. We also used word frequencies from the entire human genome as a separate control. N{*mut*|*pos*, *W*} and N{*mut*|*pos'*, *W'*} are the numbers of observed mutations in the {*mut*|*pos*, *W*} context and {*mut*|*pos'*, *W'*} subcontext, respectively.

The ratio P_*W*_/P_*W' *_estimates the probability for *W' *to be extended to *W*. This ratio coincides with the expected ratio N{*mut*|*pos*, *W*}/N{*mut*|*pos'*, *W'*} under the hypothesis that mutations rates are the same in the context {*mut*|*pos*, *W*} and its subcontext {*mut*|*pos'*, *W'*}. Therefore, if Contrast ({*mut*|*pos*, *W*}, {*mut*|*pos'*, *W'*}) is greater than 1, it indicates an increased mutation rate in the context {*mut*|*pos*, *W*} compared with the subcontext {*mut*|*pos'*, *W'*}; while if Contrast ({*mut*|*pos*, *W*}, {*mut*|*pos'*, *W'*}) is less than 1, it indicates a decreased mutation rate.

### Minimal contrast

For a given context {*mut*|*pos*, *W*} let us consider all of its subcontexts {*mut*|*pos'*, *W'*}. The minimal contrast is the value MC = Contrast ({*mut*|*pos*, *W*}, {*mut*|*pos'*, *W'*}) such that the absolute difference |MC - 1| is the lowest among all subcontexts {*mut*|*pos'*, *W'*}. We did not study discontigous contexts.

### Mutation bias

For any context {*mut*|*pos*, *W*} there exist only one subcontext {*mut*|*pos'*, *W'*} such that the length of *W' *is equal to 1 (i.e., *W' *is the one-letter word, consisting of the mutated letter). The mutation bias is the contrast of the given context and this subcontext.

### Statistical significance

The statistical significance of the hypothesis that there is an excess of mutation *mut *in the context {*mut*|*pos*, *W*} compared with its subcontext {*mut*|*pos'*, *W'*} can be computed using a binomial distribution with the following parameters:

The significance level was set to 10^-15 ^taking into account the Bonferroni correction for multiple comparisons. There were a total of 13728 multiple comparisons of context and subcontext pairs.

## Competing interests

The authors declare that they have no competing interests.

## Authors' contributions

AP, SM, AA, SS, and YP have participated equally in the design of the study, data preparation, and drafting the manuscript. AA performed the statistical analysis. All authors read and approved the final manuscript.
